# Competency (and Beyond): Think Patient. Collaborate. Develop. Evaluate. Improve. Repeat.

**DOI:** 10.3390/pharmacy7040146

**Published:** 2019-11-04

**Authors:** Andreia Bruno-Tomé, Maja Ortner Hadžiabdić, Iva Mucalo, Timothy Rennie

**Affiliations:** 1 Faculty of Pharmacy and Pharmaceutical Sciences, Monash University, Melbourne, 3052 Victoria, Australia; 2 Faculty of Pharmacy and Biochemistry, University of Zagreb, Zagreb, 10000 Zagreb, Croatia; mortner@pharma.hr (M.O.H.); imucalo@pharma.hr (I.M.); 3 School of Pharmacy, University of Namibia, Windhoek, Private Bag 13301, Namibia; trennie@unam.na

**Keywords:** competency-based, competency framework, pharmacy education, professional development, interprofessional

## Abstract

The global workforce needs to be competent, flexible, adaptable, sustainable, and patient-focused. A competency approach towards education, development, and professional practice strengthens services and increases better health outcomes. This paper will provide a global perspective on competency approaches from different health care professions. It will focus on two case reports, describing the use of competency methodologies at an undergraduate and postgraduate level, as well as supporting the internship and/or pre-registration training. Challenges and opportunities will be highlighted by addressing some of the key questions posed for this special edition.

## 1. Introduction

The Declaration of Astana [1] by the World Health Organization (WHO) urges society to take action towards the Universal Health Coverage (UHC) and the Sustainable Development Goals [2]. It outlines the commitment to the fundamental right of UHC by, among others, building a sustainable primary health care (PHC), which includes capacity building as one of the main drivers for success. It also includes a call to action from all stakeholders to align with national policies, strategies, and plans across all sectors through people-centred and gender-sensitive approaches in order to achieve a sustainable PHC.

The Global Health Workforce Network (GHWN), a network that operates within WHO, created several Hubs to support the implementation of the Global Strategy on Human Resources for Health: Workforce 2030 [3]. The Education Hub has set as priority the development of a Global Competency Framework for UCH, aligning the needs from student selection all the way to the health labour market needs. The framework will focus on the competencies and activities of health professionals and health associate professionals at the foundation level, including the pharmaceutical workforce [4].

Across professions, competency-based approaches or competency development frameworks—containing behavioural competencies (knowledge, skills, attitudes, and behaviours), grouped into domains/clusters—have become increasingly accepted in education, driven by the need for clarity in the training, development, and professional recognition of health care professionals [5,6,7,8,9]. Across all frameworks or documents, competencies such as communication, patient centred care, teamwork, professionalism, health advocacy, management, and leadership are at the core for the development of the health workforce, to improve the delivery of services, and ultimately, the patients’ outcomes.

Competency is no stranger concept to the pharmacy profession and it may have many meanings [10]. In 2012, the International Pharmaceutical Federation (FIP) developed the Global Competency Framework (GbCF) for services provided by pharmacy workforce [11] based on education developmental frameworks for pharmacists to aid its members to navigate this complex concept. It is intended to act as a mapping tool and can be adapted according to the country or local needs. The GbCF is divided into domains, competencies, and behavioural competencies. Several countries have used it as base for their own frameworks, including Chile, Croatia, Jordan, Serbia (including collaborating with other health care professionals), Thailand, and the UK. How it was developed and its impact is briefly described in the FIP Transforming Our Workforce report [12]. The Pharmaceutical Society of Ireland (PSI) is another example, illustrating also that a competency framework has a number of purposes from curriculum development and pre-registration programmes, continuing professional development (CPD), development of specialisation, and advanced practice, as well as a benchmark for the society by state the professional role of a pharmacist [13].

This paper aims to illustrate with two case reports, where competency is at the core of pharmacists’ development, considering different aspects that need evaluation and focus. Case 1 focuses on the use of a competency framework to design a pre-registration training programme for undergraduate pharmacy students. Case 2 provides an overview of how a need for in-country pharmacy training, meant the development of new training programmes—at undergraduate and postgraduate level—to ensure that graduates are competent to meet the health needs of the population.

Challenges, opportunities, and what to do next will be answered in this special issue—Pharmacy Education, Competency, and Beyond.

## 2. Case 1: Utilization of Competency Framework in Undergraduate Education in Croatia

### 2.1. Introduction

In the academic year 2011/2012, a Croatian pharmacy study program was harmonized with the Directive 2005/36/EC which stipulated a “six-month traineeship in a pharmacy open to the public or in a hospital”. According to the revised curriculum at the Faculty of Pharmacy and Biochemistry University of Zagreb (UoZ), one-year pre-registration training for undergraduate pharmacy students has been replaced with the six-month Professional Training for Pharmacists (PTP).

PTPs’ program was designed based on the Croatian Competency Framework (CCF), a validated tool for assessment and self-assessment of community and hospital pharmacists. It comprises a list of behaviours and competencies that need to be adopted in the pre-registration period. The competence-based approach in education enables learning in a way that clearly connects learning and work. Implementation of competency-based training helps to produce graduates equipped to respond capably in a rapidly changing environment.

The aim of this case was to demonstrate the application of the CCF in designing the pre-registration training program for undergraduate pharmacy students. Furthermore, it describes the use as the evaluation tool for students’ and pharmacist-preceptors’ competencies development during PTP.

### 2.2. Methodology

The expert group for designing the PTPs’ program at the UoZ Faculty of Pharmacy and Biochemistry were approached using the convenience sampling methodology and consisted of nine members [academia (six), community pharmacists (three)]. The expert group explored and discussed various approaches for pre-registration programme design and reached a consensus on employing the CCF as a core document. Details on the CCF, a previously validated competency framework published [14]. From the beginning of the data-reviewing process, an iterative process of 10 rounds of consensus development panels in the period from February 2015 to September 2015 was undertaken to evaluate and make changes to the initial competency framework, namely CCF.

Subsequently, all students (n = 126) attending PTP in the period of January 2016 to March 2017 were asked to self-assess their competencies in order to monitor their progress. Students self-assessed themselves by using the adjusted version of the CCF at two time-points at the beginning (following two to four weeks) and at the end (following six months) of the PTP. Self-assessment was performed on a four-point Likert scale indicating how often they demonstrated each behaviour, i.e., “rarely” (0–24% of time), “sometimes” (25–50% of time), “usually” (51–84% of time), and “consistently” (85–100% of time).

Additionally, community pharmacists-preceptors, who have voluntarily accepted the invitation of the UoZ Faculty of Pharmacy and Biochemistry to provide support and mentoring to students-trainees within the PTP, also assessed their competencies on a four-point Likert scale by using the CCF [15]. To ensure a high response rate, a mixed-mode approach of data collection that combined two rounds of e-mailed survey instrument with a telephone reminder was employed in the period from October 2015 to April 2016.

### 2.3. Results and Discussion

The CCF was adjusted for the PTPs’ program by a consensus method and was organized into 16 competencies and 68 behaviours, thus rendering 70% of competencies from the original CCF. Some of the competencies from the original CCF were deemed too complex or inessential to be demonstrated at the undergraduate level and were therefore excluded (e.g., develop, implement and conduct Standard Operating Procedures) or assigned a lower level of activity (i.e., level of understanding instead of demonstrating a behaviour). Additionally, the Expert group developed specific competency-based students’ tasks that need to be completed during the PTP with an aim of guiding their competency development.

Following the development of competency-based pre-registration program, adapted CCF was used as a self-assessment tool by 126 students, predominantly female (n = 110, 87.3%), with a median age of 24 (23–27) years. Both at the beginning and at the end of the PTP, students consistently demonstrated behaviours pertaining to the “Personal and Professional competencies” cluster (2.92 ± 0,516 and 3.40 ± 0.439, respectively), whereas they rarely demonstrated behaviours in the “Pharmaceutical Public Health Competencies” cluster (1.94 ± 0.741 and 2.93 ± 0.705, respectively). During the six-month pre-registration training, students’ development of all CCF competencies was significant (*p* < 0.001) with the greatest improvements seen in the following competencies: health promotion, patient consultation, and assessment of medicines and medical devices.

Additionally, CCF was used for pharmacists-preceptors’ self-evaluation [15]. Of the 260 community pharmacists approached, a final analysis included 223 respondents. The response rate was 85.8%. Community pharmacist-preceptors assessed themselves as the most competent in competencies pertaining to the cluster “organization and management competencies” (3.64 ± 0.34), while they considered themselves as the least competent in the competencies pertaining to the cluster “pharmaceutical public health competencies” (2.75 ± 0.77).

This research identified students and community pharmacist-preceptor competencies that require improvement. Consequently, areas for additional professional education were defined. Implementing modalities to measure and support development of preceptors’ competences are essential for improvement of student training programmes.

### 2.4. Conclusion

The experience and the evidence obtained revealed that the adapted General Level Competency Framework, namely CCF, can be utilized in the PTPs’ program design for final year pharmacy students in Croatia. Moreover, it proved to be useful as the assessment and progress monitoring tool for both students and pharmacists-preceptors, thus providing a link between training and practice and underpinning the competency-led undergraduate training program. It remains to compare the competency-based assessment with other standard assessment methods [written and oral exams, OSCE (Objective Structured Clinical Examination)]. The recommendation is to gather such data as to conclude whether the competency assessment should be used exclusively or in conjunction with other assessment methods.

## 3. Case 2: A Modern Curriculum for Namibia by Defining the Needs and Articulating a Competency Framework

### 3.1. Introduction

Namibia, a post-apartheid country in southern Africa that gained independence in 1990s, has struggled to deliver equitable health care in a setting with low population density, high income inequality, and the health burdens of HIV, tuberculosis, and growing non-communicable diseases. Furthermore, health care access has been thwarted by scant healthcare professionals [16], such that the government invested heavily in providing in-country pharmacy training as well as other health cadres for the first time through the University of Namibia (UNAM).

The focus, therefore, has been to develop new training programmes at undergraduate and postgraduate level as well as supporting the internship/pre-registration training, and to maintain a level of quality that will ensure graduates are competent to meet the health needs of Namibia. The vision of the UNAM School of Pharmacy has been to prioritise the training needs to provide a competent workforce in the pharmacy profession. For example, by also training Pharmacy Technicians to work alongside pharmacists, career pathways for progression that is based on improving competence through training, as well as an interprofessional emphasis to maximise population health outcomes. The Bachelor of Pharmacy curriculum, for example, was developed by defining the needs [17], articulating a competency framework, and in turn extrapolating a modern curriculum [18].

Now having successfully gained national accreditation, and with over five graduate classes entering practice, the intention is to measure the impact on care by using work-based assessments that are integrated through postgraduate training programmes (currently being piloted). In this setting, competence of graduates must be linked with the needs of the country that is likely to be partially, but not entirely, mapped by global competency frameworks. For example, a significant role for pharmacists in Namibia in a large sparsely populated country with very limited industry is in supply chain management to meet the medicines needs even in the event of heavy flooding giving rise to unusually high malaria levels.

### 3.2. Discussion

As pharmacy training at undergraduate, technical, and postgraduate (clinical pharmacy) levels are relatively new in Namibia, there are a broad range of indicators that are being developed and assessed. These relate not just to competence of graduate pharmacists in practice, but also aspects such as training quality (are pharmacy students able to graduate, register and practice?), workforce development (are graduates getting employment and in what sectors? Is this meeting population needs?), pharmacy-related infrastructure and industry (are new pharmacy premises being created? New/better supply chain, industrial manufacturers, quality control laboratories?), and professional cohesion (how well are students and graduates working together within the profession and with other professions to improve patient care?). A number of projects are currently being undertaken to quantify this impact (Table 1).

Data from the competence of graduates is limited due to the fact that all the training programmes are relatively new [19,20,21]. The School is currently planning research to measure the impact of new programmes in Namibia and the competence of pharmacists therein, but will take some time to report on.

### 3.3. Conclusions

Although the situation in Namibia is somewhat unique, any assessment of workforce competence arguably needs to be mapped against the broader landscape in any given setting. It is likely that simply increasing the volume of the workforce at service-entry level will have the most significant impact on population health outcomes and any additional improvements will be incremental (the law of diminishing returns).

In Namibia, the workforce has grown at least three-fold since the original needs-assessment was conducted to investigate the feasibility of in-country training. Nonetheless, providing a more competent workforce is also likely to have sizeable and measurable impact, although it will be important to systematically and concurrently measure aspects, such as, workforce cohesion. Are population patient outcomes improving because the pharmacy workforce is more competent or because they are working better together? Is this an aspect of competence? Time will tell whether it will be possible to better measure and understand the impact of workforce competence in Namibia. This will have an impact on the type of (new) training offered as well as the content.

Pharmacy training in Namibia has now been underway for eight years with massive changes observed. In another decade, the landscape could be very different and will require an informed, ongoing analysis of indicators, including competence to plan for the future.

## 4. Challenges and Opportunities

From the two case reports, one can argue that there will always be challenges and opportunities when it comes to developing competency-based approaches. The chosen cases demonstrate the different approaches taken to reach a common goal—increase better health outcomes for the patient through a competent workforce. The drivers for change can differ in these two cases: one from a policy directive approach, and the other from a health needs assessment, nonetheless using the same core concept—competency development.

Challenges can relate to financial and technological characteristics of the countries or settings as well as the overall sustainability of project. Opportunities can be seen as a way to improve PHC, increasing the perceived impact on patients’ outcomes and ultimately addressing the UHC.

### 4.1. Can Academic and Scholarly skills and Standards Continue to Be Met in Professional Programmes?

The Croatia case demonstrated that by including in the expert panel, partners from practice the competency framework developed was a useful assessment and monitoring tool for both students and pharmacists-preceptors. It also allowed pharmacists-preceptors to identify competencies that required improvement, leading to the development or educational programmes for the current pharmaceutical workforce.

### 4.2. How Can Supervision, Assessment and Feedback Be Provided Effectively and Efficiently in Experiential Placements?

Competency frameworks have been proven useful as an educational tool. In the case of Croatia, implementing techniques to measure and support the development of pharmacists-preceptors’ competencies were essential for the improvement of the experiential placement programme.

### 4.3. Are There Other Aspects of Learning and of Professional Practice That Should Be Evaluated?

The Namibia case illustrate the answer to this question by considering several research domains that need to be evaluated to quantify the impact of the training being conducted, including infrastructure and professional cohesion (introducing interprofessional learning scale).

### 4.4. Should the Focus of Assessment Be the Individual, the Team, Patient Reported Outcomes or the Programme?

Considering the pharmaceutical workforce with all of its components, as the case of Namibia, by including the training of pharmacy technicians as an example to work alongside pharmacists, creates an emphasis on interprofessional overall competence to maximise health outcomes.

### 4.5. Should the Move Be Toward a Patient Centred Approach, or Even a Conditions Approach?

Aligned with cases exemplified in this paper, which considered a pharmacy team approach, there is a need to move education towards a broader “team” approach to train more effective health professionals to work together. It is not only crucial, but essential [22]. The WHO publication—*Preparing a health care workforce for the 21st century: The Challenge of Chronic Conditions*—calls for transforming the health care workforce to better meet the needs of the population and to enhance the skills of the health professionals to meet new complexities [23]. A set of core competencies for patients with chronic conditions, endorsed by health care professions such as the World Medical Association, the International Council of Nurses, the International Pharmaceutical Federation, the European Respiratory Society, and the international Alliance of Patient’s Organizations seems to be indicative that the focus should be on patient outcomes that can be reached by a team of healthcare professionals working towards a common goal.

### 4.6. What is Next?

It is essential to consider what are the needs that must to be met. To engage with in-country stakeholders (including academia and all practice settings), and if possible, aligned with international targets [24,25,26]. There is a need to collaborate with health care professionals and non-health care professionals (when applicable and depending on the need that is being addressed). Implementation tools that have multiple purposes need to be developed. It must be evaluated if what has been created is supporting the development of the workforce. Ultimately, improve what has been created, the need for periodic evaluation and adaption ensures contemporaneity.

## 5. Conclusions

Competency has been evolving in the last few decades and is here to stay. It has proven its value in the development, enhancement, and sustainability of the pharmaceutical workforce. Ensuring a competent pharmaceutical workforce is crucial for optimising health outcomes. Ongoing analysis and evaluation of current approaches will always be necessary, as there is no one model that is appropriate to all. Collaboration across professions will create a flexible and adaptable workforce that can meet the challenges of healthcare. For the future: think patient. Collaborate. Develop. Evaluate. Improve. Repeat.

## Figures and Tables

**Table 1:** Measuring success of in-country pharmacy training in Namibia.

Research Domain	Example Indicators	Existing Projects/Research
Competence level	Work-based assessments Peer-reported feedback Impact of clinical interventions	Audit of work-based assessment performance during clinical training Audit of pharmacy interventions log Comparing competence between Namibia and other settings
Training output quality and relevance	Number of new training programmes Number of graduates Perceived relevance of training in practice domains Accreditation status	Undergraduate and graduate surveys Graduate and employer survey of relevance of pharmacy curricula
Workforce	Growth of registered pharmacy professionals (numbers, cadres) Sectoral distribution Career and training intentions New positions/roles created by sector	Quantifying career intentions to plan future training National workforce survey and observatory creation
Infrastructure	Number of new pharmacy premises and industry sites Medicines shortages	Ongoing medicines shortage survey and observatory creation
Professional cohesion	Proportion of pharmacy staff integrated in pharmacy-based or clinical teams Healthcare worker perceptions	Ongoing ‘readiness for interprofessional learning scale’ survey (healthcare students)
